# The effect mechanism of perceived entrepreneurial environment on Chinese college students’ entrepreneurial intention: chain mediation model test

**DOI:** 10.3389/fpsyg.2024.1374533

**Published:** 2024-06-26

**Authors:** Qicheng Lin, Yirong Chen, Junli Lai, Xinyi Zhang

**Affiliations:** School of Educational Science, Quanzhou Normal University, Quanzhou, China

**Keywords:** college students, perceived entrepreneurial environment, achievement motivation, entrepreneurial self-efficacy, entrepreneurial intention

## Abstract

**Introduction:**

This study aimed to explore the effect of perceived entrepreneurial environment among Chinese college students’ entrepreneurial intention and its underlying mechanism.

**Methods:**

Based on a survey of 445 college students from 5 universities with the perceived entrepreneurial environment assessment scale, the achievement motivation scale, the entrepreneurial self-efficacy scale, and the entrepreneurial intention questionnaire.

**Results:**

There were significant correlations among perceived entrepreneurial environment, achievement motivation, entrepreneurial self-efficacy, and entrepreneurial intention, and perceived entrepreneurial environment could significantly positively predict entrepreneurial intention. Achievement motivation and entrepreneurial self-efficacy played significant mediating roles between the perceived entrepreneurial environment and entrepreneurial intention. There were three paths that perceived entrepreneurial environment to influence entrepreneurial intention: One was the mediating role of achievement motivation; The second was the mediating role of entrepreneurial self-efficacy; The third was the chain-mediated role of both achievement motivation and entrepreneurial self-efficacy.

**Discussion:**

The internal mechanism of the relationship between perceived entrepreneurial environment and entrepreneurial intention enriches the research results of entrepreneurial psychology among college students and provides a theoretical basis for training and guiding the entrepreneurship of college students.

## Introduction

Entrepreneurial intention is a critical initial step in the entrepreneurial process. It refers to an individual’s conscious plan to pursue entrepreneurial activities and is an important predictor of entrepreneurial behavior (Krueger et al., 2000; Fan and Wang, 2006; Thompson, 2009). Entrepreneurship can not only increase the employment of undergraduates and promote sustainable socio-economic growth, but also bring market innovation, improve economic efficiency, and alleviate social tensions (Li and Zhang, 2015; Chang and Lyu, 2022). As a result, it has become an important career choice among Chinese college students. Existing research mainly focuses on the influencing factors of entrepreneurial intention from a certain aspect such as entrepreneurial ability and entrepreneurial environment, but there is insufficient attention paid to the systematic influencing factors of entrepreneurial intention and its mediating effects (Ankita and Parwinder, 2024). In addition, the academic community has not paid enough attention to the specific group of college student entrepreneurs, mainly because different scholars have different research perspectives. Previous studies have focused more on the internal and external factors of entrepreneurs (Mohammad and Majed, 2024). In fact, the influencing factors of entrepreneurial intention among college students are diverse, driven by both internal and external factors (Lyu et al., 2024). Therefore, this study systematically investigates the influencing factors of college students’ entrepreneurial intention guided by the perception of entrepreneurial environment, and reveals the theoretical and practical value of college students’ achievement motivation and entrepreneurial self-efficacy in their entrepreneurial growth process. It can also provide theoretical guidance and empirical basis for guiding and encouraging college students to start businesses.

The models of entrepreneurial intentions argue that perceived entrepreneurial environment is one of the important factors influencing individual entrepreneurial acts (Lüthje and Franke, 2003). Perceived entrepreneurial environment is the sum of a range of external factors perceived by individuals that influence the emergence and development of the entrepreneurial activity, including regulatory, normative, and cognitive factors (Wei, 2015). Studies have confirmed that individuals are less likely to have entrepreneurial intentions when they perceive that environmental factors from school, family, and society hinder entrepreneurial acts (Zhao and Du, 2016). However, individuals will increase their entrepreneurial intention when they perceive supportive entrepreneurial environments. Based on this, this study hypothesized 1 that the perceived entrepreneurial environment is an important factor affecting entrepreneurial intention. Besides, social cognitive theory suggests that individual behavior changes with changes in both individual and environmental factors. Entrepreneurial intention is affected not only by an individual’s perception of the objective environment (entrepreneurial environment assessment) but the intrinsic psychological dynamics of the individual (the achievement motivation) and the belief that the individual is competent for the role or complete the task (the entrepreneurial self - efficacy). Therefore, this study will investigate how perceived entrepreneurial environment acts on entrepreneurial intention through internal psychological variables (achievement motivation) and individual characteristic variables (entrepreneurial self-efficacy).

The expectation-value theory argues that when individuals are in a competitive environment, they experience two psychological tendencies: the motivation to pursue success and the motivation to avoid failure (Atkinson, 1957). When the motivation to pursue success is greater than the motivation to avoid failure, individuals tend to have a higher estimation of success goals and work hard to achieve them. Achievement motivation, an important internal trait, is the intrinsic motivation that drives individuals to pursue meaningful, valuable tasks and seek to acquire (Mcclelland, 1961). The formation of achievement motivation is the result of the interaction between individuals and their environment. It has been confirmed by studies that a good entrepreneurial environment is an important factor that can significantly predict individual achievement motivation (Yang et al., 2019). Therefore, perceived entrepreneurial environment can predict individual achievement motivation significantly and positively. On the other hand, it has been founded by studies that entrepreneurs often have a strong motivation for achievement. It is because that entrepreneurship is different from ordinary employment, when individuals have a strong motivation for achievement, the more courageous they are to take risks to start a business, the stronger their willingness to start a business. According to the theory of planned behavior (Ajzen, 1991), behavioral intention is the most direct factor influencing behavior, and attitude and motivation play a key role in the influence of individual behavior. It also has been confirmed by studies that individual achievement motivation can significantly and positively predict entrepreneurial intention (Brandstatter, 2011). Perceived entrepreneurial environment may influence entrepreneurial intention through the mediating role of achievement motivation. Therefore, based on the relationship between perceived entrepreneurial environment, entrepreneurial self-efficacy, and entrepreneurial intention, hypothesis 2 of this study is that achievement motivation is the mediating variable between entrepreneurial environment assessment and entrepreneurial intention.

Entrepreneurial self-efficacy is the belief that an individual will be successful in the role of an intrapreneur or complete the tasks of intrapreneurship (Chen et al., 1998). The social cognitive theory argues that the relationship between individuals, cognition, and environment in the social environment constitutes a dynamic interaction. The individual’s analysis of tasks, attribution of past successes and failures, and evaluation of the environment affect the formation of self-efficacy (Bandura, 1997). Entrepreneurial self-efficacy is based on the assessment of the entrepreneurial environment. When individuals feel the support of an objective environment, it will enhance their sense of security, eliminate the fear of failure, and promote the formation of entrepreneurial self-efficacy (Hopp and Stephan, 2012). Studies have confirmed that the perception of an entrepreneurial environment composed of individual growth experience and social support can significantly predict entrepreneurial self-efficacy and influence entrepreneurial behavior (Arias et al., 2021). On the other hand, according to Bandura’s theory of self-efficacy, self-efficacy is the decisive factor of individual behavior. It is the psychological driving force for the continuous regulation of individual behavior. Entrepreneurial self-efficacy is considered a key predictor of individual entrepreneurial intention and ultimately entrepreneurial action. It plays a key role in the formation and development of individual entrepreneurial intention (Wang et al., 2023). Numerous studies have confirmed that higher entrepreneurial self-efficacy leads to the greater conviction that one’s own entrepreneurship is feasible, and can significantly predict an individual’s entrepreneurial intention, behavior, and performance (Wilson et al., 2010; Su and Qiao, 2019). In view of the above, hypothesis 3 of this study is that entrepreneurial self-efficacy is the mediating variable between perceived entrepreneurial environment and entrepreneurial intention.

The achievement motivation is closely related to entrepreneurial self-efficacy (Li and Zeng, 2018), and it positively predicts entrepreneurial self–efficacy (Wang and Yu, 2023). College students with higher achievement motivation have stronger self-learning ability, career adaptability, entrepreneurial ability, and career maturity, as well as a higher sense of entrepreneurial self-efficacy (Wu et al., 2016). Therefore, the impact of achievement motivation on entrepreneurial intention may be realized through entrepreneurial self-efficacy. Based on this, hypothesis 4 of this study is that perceived entrepreneurial environment can influence entrepreneurial intention through the chain mediating effect of achievement motivation and entrepreneurial self-efficacy.

Above all, this study intends to systematically investigate the effects of perceived entrepreneurial environment, achievement motivation, and entrepreneurial self-efficacy on entrepreneurial intention, as well as the mediating effects of the entrepreneurial achievement motivation and entrepreneurial self-efficacy in the relationship between perceived entrepreneurial environment and entrepreneurial intention. Finally, it reveals how perceived entrepreneurial environment affects entrepreneurial intention. Considering that previous studies have found that the gender, grade, family economic status are significantly related to entrepreneurial intention. Therefore, these variables were controlled for in this study.

## Measures

### Participants

Using a convenient sampling method, 469 Chinese college students from five universities were selected to participate in the survey from February to June 2023. The collected data were screened, and 445 valid questionnaires were collected, with an effective rate of 94.9%. The sample included 147 (33.0%) male and 298 (67.0%) female; 162 (36.4%) freshmen, 136 (30.6%) sophomores, 77 (17.3%) juniors and 70 (15.7%) seniors; 313 (70.3%) were from rural areas and 131 (29.4%) were from urban areas. There were 8 (1.8%) students from economically poor families, 44 (9.9%) students from lower-middle families, 339 (76.2%) students from middle-level families, 52 (11.7%) students from upper-middle families, and 2 (0.4%) students from affluent families. In terms of academic disciplines, 200 (44.9%) participants were from liberal arts, 61 (13.7%) were from science, 22 (4.9%) were from engineering, 49 (11%) were from arts, 37 (8.3%) were from business subjects, 6 (1.3%) were from physical education, and 70 (15.7%) were from other subjects. The average age of the participants was 19.85 (*SD* = 1.89).

### Measures

#### Perceived entrepreneurial environment assessment scale

The Perceived Entrepreneurial Environment Assessment Scale prepared by Huang et al. (2020) was used, which was compiled on the basis of Wei Hongmei’s entrepreneurial environment theory. This study is revised appropriately according to the actual situation of college students. For example, the second question “The school’s leaders are very supportive of the teachers’ entrepreneurial activities” is changed to “The teachers are very supportive of our entrepreneurial activities”; the question “My colleagues have supported me in entrepreneurial activities and provided the necessary help” is changed to “my teachers have supported me in entrepreneurial activities and provided the necessary help”; the question “when I participate in various forums, my contacts or discussions with my colleagues can provide me with useful entrepreneurial information and entrepreneurial skills” is changed to “my contacts or discussions with my friends can provide me with useful entrepreneurial information and entrepreneurial skills.” The scale is divided into three dimensions with a total of 8 questions, namely, micro-regulatory environment assessment (such as “the school provides us with services such as an entrepreneurial platform”) reflects the school’s support for the entrepreneurial system, and micro-normative environmental assessment (such as “my family members have supported me in entrepreneurial activities and provided the necessary help”) reflects the atmosphere and effective action support of social encouragement and support for entrepreneurship. Micro-cognitive environmental assessment (such as various industry conferences, academic conferences, workshops, and courses that help me acquire the information and skills I need to start a business) examines the knowledge and skills to acquire entrepreneurship. A score of 5 points is used, with 1 indicating complete disagreement and 5 indicating full agreement, and higher scores indicating a better perceived entrepreneurial environment. The measurement model fit index is *χ^2^*/*df* = 2.50, GFI = 0.98, AGFI = 0.95, NFI = 0.97, IFI = 0.98, TLI = 0.96, CFI = 0.98, RMSEA = 0.06. The Cronbach’a coefficient for the total questionnaire in this study was 0.83.

### Achievement motivation scale

The Achievement motivation scale revised by Ye and Hagtvet (1992) was used to test the subjects. A total of 30 items, including motive to achieve success (Ms), for example, “I like to work tirelessly on problems that I am not sure about solving.” and motive to avoid failure (Mf), for example, “I hate working in situations where I’m completely unsure if I’ll fail.” A score of 4 points is used, with 1 indicating complete disagreement and 4 indicating full agreement, Mt. = Ms. – Mf. The measurement model fit index is *χ^2^*/*df* = 2.49, IFI = 0.91, TLI = 0.90, CFI = 0.91, RMSEA = 0.06. The Cronbach’a coefficient for the total questionnaire in this study was 0.93.

### Entrepreneurial self-efficacy scale

The Entrepreneurial self - efficacy scale compiled by Li (2017) was used to test the subjects. The scale consists of 6 dimensions and 32 items. They are opportunity recognition effectiveness (such as I am able to respond promptly to business opportunities’), leadership effectiveness (such as I am able to articulate the company’s vision and values), human resource management effectiveness (such as I am able to persuade others to accept my perspective ‘), product innovation effectiveness (such as I am able to identify new areas with high growth potential), willpower efficacy (such as I am able to respond quickly to unexpected changes and failures’) and risk tolerance efficacy (such as I am able to work efficiently in a continuously high-pressure work environment ‘). The scale uses a 5-point rating, from “1″ (completely inconsistent) to “5″ (completely consistent). A score of 5 points is used, with 1 indicating complete disagreement and 5 indicating full agreement, and higher scores indicating a better perceived entrepreneurial environment. The measurement model fit index is *χ^2^/df* = 3.10, IFI = 0.92, TLI = 0.90, CFI = 0.91, RMSEA = 0.07. The Cronbach’s a coefficient for the total questionnaire in this study was 0.95.

### Entrepreneurial intention questionnaire

The entrepreneurial intention questionnaire compiled by Gelderen (2006) and revised by Li et al. (2008) was used to test the subjects. The scale has 5 items in total. The first four questions are “I think I will start a business in the future,” “I have considered running my own company,” and “If I have the opportunity and am free to make decisions, I will choose to start my own business,” “Considering my current situation and various limitations (such as lack of funds), I will still choose to start my own business,” using a 5-point scoring system, from “1” (completely disagree) to “5” (completely agree), The fifth question, “What do you think is your likelihood of starting a business in the next 5 years?” uses a percentage score, ranging from “1” (0%) to “5” (100%). The higher the total score, the stronger the willingness to start a business. The measurement model fit index *χ^2^*/*df* = 2.16, GFI = 0.99, AGFI = 0.97, NFI = 0.99, IFI = 0.99, TLI = 0.99, CFI = 0.99, RMSEA = 0.05. The Cronbach’s *a* coefficient for the total questionnaire in this study was 0.90.

## Procedure

After obtaining informed consent from the school and individuals, professional teachers distributed paper questionnaires to the participating students, uniformly introduced the guidelines, and informed them of the testing content and requirements. The questionnaire is anonymous to ensure the authenticity and reliability of the survey. The testing time is about 15 min, and all questionnaires are collected on-site. This study received funding from the first author’s institution for scientific research and innovation, and was approved by the school’s review committee. After the test, participants can receive a gift of one dollars as a reward.

### Analyses

In the present study, the missing value data in the participants’ responses were less than 5%, so no data were deleted. We used SPSS 24.0 and Amos version 24.0 for data analyses. The data were sorted and statistically analyzed using SPSS version 24.0, and Amos version 24.0 was used for confirmatory factor analysis. First, descriptive analyses and Pearson correlation analyses were conducted to report means, standard deviations, and correlations for the interested variables. Secondly, the mediation effect was calculated using SPSS 24.0 with Hayes’s PROCESS windows (Hayes, 2017) to further explore the relationship of the interest variables.

## Results

### Common method bias

In this study, anonymous tests and reverse-scoring questions were used to control the common method bias. We used Harman’s one-factor test (Podsakoff et al., 2003) to determine whether the data exist common method. In this test, we used the SPSS factor analysis routine to identify the first eigenvalue from the data matrix. The test results reveal that the first eigenvalue accounts for 24.71% of total variances, which does not equate to the majority of the total variance explained (threshold of 40%). Thus, according to Harman’s one-factor test, common method bias is not likely to bias the results (Zhou and Long, 2004).

### Descriptive statistics

The descriptive statistics and results of the correlation analyses of the main variables in this study are shown in Table 1. It was found that the perceived entrepreneurial environment was positively correlated with achievement motivation (*r* = 0.43), entrepreneurial self-efficacy (*r* = 0.58), and entrepreneurial intention (*r* = 0.54). Achievement motivation was positively correlated with entrepreneurial self-efficacy (*r* = 0.60) and entrepreneurial intention (*r* = 0.43). Entrepreneurial self-efficacy was positively correlated with entrepreneurial intention (*r* = 0.48).

**Table 1:** Correlations, means, and standard deviation among study variables.

	*M*	SD	1	2	3	4	5	6	7
1.Gender	0.33	0.47	—						
2.Grade	2.12	1.07	−0.02	—					
3.Family economic status	2.99	0.55	0.01	−0.01	—				
4.Perceived entrepreneurial environment	3.38	0.55	0.06	−0.12	0.16^***^	—			
5.Achievement motivation	0.04	0.89	0.16^***^	0.06	0.13^**^	0.43^***^	—		
6.Entrepreneurial self-efficacy	3.66	0.56	0.06	0.01	0.16^**^	0.58^***^	0.60^***^	—	
7.Entrepreneurial intention	2.50	0.93	0.18^***^	0.03	0.12^*^	0.54^***^	0.43^***^	0.48^***^	—

*N* = 445; Gender is a dummy variable, with females = 0 and males = 1, and the mean represents the proportion of males. Grade is a dummy variable, freshman = 1, sophomore = 2, junior = 3, senior = 4. Family economic status is a dummy variable, poverty = 1, lower middle = 2, average = 3, upper middle = 4, wealth = 5. ^*^p < 0.05; ^**^p < 0.01; ^***^p < 0.001.

### Mediating effect analysis

**Figure 1:**
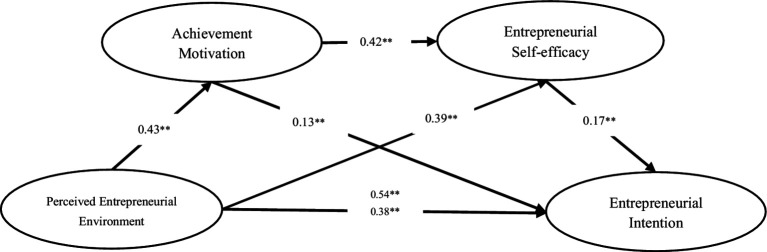
Hypothesized model.

After controlling for gender, grade, and family economic status, the mediating model analysis results show that the path coefficients of gender, grade, and family economic status were not significant. we examined the direct pathway of the perceived entrepreneurial environment to entrepreneurial intention. The results showed that the perceived entrepreneurial environment positively predicted entrepreneurial intention (*β* = 0.54, *p*<0.001). Then, the mediation model was reanalyzed. The results of the mediating effect of achievement motivation and entrepreneurial self-efficacy are shown in Figure 1. The results showed that perceived entrepreneurial environment had a significant positive effect on achievement motivation (*β* = 0.43, *p* < 0.001), while burnout had a significant positive effect on entrepreneurial self-efficacy (*β* = 0.39, *p* < 0.001). Achievement motivation had a significant positive effect on entrepreneurial self-efficacy and entrepreneurial intention (*β* = 0.42, *p* < 0.001; *β* = 0.13, *p* < 0.01), respectively. And entrepreneurial self-efficacy had a significant positive effect on entrepreneurial intention (*β* = 0.17, *p* < 0.01). In addition, the perceived entrepreneurial environment had a significant positive effect on entrepreneurial intention (*β* = 0.38, *p* < 0.001).

The bootstrap 95% CI confirms the significant indirect effects of achievement motivation and entrepreneurial self-efficacy in the relationship between perceived entrepreneurial environment and entrepreneurial intention (Table 2). These results indicate that achievement motivation and entrepreneurial self-efficacy not only partially mediate the relationship between perceived entrepreneurial environment and entrepreneurial intention, but also have a chain mediating effect on them (Table 2 and Table 3).

**Table 2:** Results of hierarchical regression analysis study variables.

	Achievement motivation			Entrepreneurial self-efficacy			Entrepreneurial intention		
	*β*	SE	*t*	*β*	SE	*t*	*β*	SE	*t*
Gender	0.29^***^	0.09	3.23	−0.06	0.07	−0.77	0.27^**^	0.08	3.37
Grade	0.10^**^	0.04	2.64	0.03	0.03	0.92	0.06	0.04	1.80
Family economic status	0.12	0.08	1.55	0.08	0.06	1.36	0.03	0.07	0.44
Perceived entrepreneurial environment	0.43^***^	0.04	10.01	0.39^***^	0.04	10.13	0.38^***^	0.05	8.08
Achievement motivation				0.42^**^	0.04	10.95	0.13^**^	0.05	2.71
Entrepreneurial self-efficacy							0.17^**^	0.05	3.19
*R*		0.47			0.70			0.61	
*R* ^2^		0.22			0.49			0.37	
*F*		31.53^***^			83.35^***^			43.26^***^	

*N* = 445; ^*^p < 0.05; ^**^p < 0.01; ^***^p < 0.001.

**Table 3:** Summary of hypotheses testing.

	Effect	Boot SE	Boot LLCI	Boot ULCI	Proportion of total
Total effect	0.16	0.03	0.09	0.23	28.48%
Indirect pathway 1	0.06	0.02	0.02	0.10	10.50%
Indirect pathway 2	0.03	0.01	0.01	0.06	5.71%
Indirect pathway 3	0.07	0.03	0.02	0.12	12.27%

Indirect pathway 1: perceived entrepreneurial environment→achievement motivation→entrepreneurial intention; Indirect pathway 2: perceived entrepreneurial environment→achievement motivation→entrepreneurial self-efficacy→entrepreneurial intention; Indirect pathway 3: perceived entrepreneurial environment→entrepreneurial self-efficacy→entrepreneurial intention.

## Discussion

### The relationship between perceived entrepreneurial environment and entrepreneurial intention

This study explores the relationship between perceived entrepreneurial environment and entrepreneurial intention based on the theory of the entrepreneurial intention model, as well as its internal mechanism. The results indicate that perceived entrepreneurial environment is significantly and positively correlated with entrepreneurial intention, it can significantly predict college students’ entrepreneurial intention, which is consistent with previous research results (Saeed et al., 2014; Du et al., 2016; Ye and Fang, 2017). Additionally, it also has a significant positive impact on promoting entrepreneurial intention. It is worth noting that after introducing the two mediating variables of achievement motivation and entrepreneurial self-efficacy, the influence of perceived entrepreneurial environment on entrepreneurial intention remained significant. The results support the theory of the entrepreneurial intention model, and perceived entrepreneurial environment is an important factor affecting college students’ entrepreneurial intention. Therefore, to enhance Chinese college students’ entrepreneurial intention, it is necessary to pay attention to the factors of the entrepreneurial environment. A good entrepreneurial environment not only provides a crucial channel for college students to access entrepreneurial resources but also stimulates their entrepreneurial passion (Zhou and Wang, 2019).

### The separate mediating effect of achievement motivation and entrepreneurial self-efficacy

The results show that perceived entrepreneurial environment can influence entrepreneurial intention through the separate mediating roles of achievement motivation and entrepreneurial self-efficacy. These results are consistent with previous studies conducted on college students in the mainland, which have found that achievement motivation mediate the relationship between perceived entrepreneurial environment and entrepreneurial intention, the entrepreneurial self-efficacy mediate the relationship between perceived entrepreneurial environment and entrepreneurial intention.

The subjective environmental system (perceived entrepreneurial environment) can only act on the individual through the individual’s internal psychological dynamic system (achievement motivation). Compared to those with a low perceived entrepreneurial environment, college students with a high entrepreneurial environment assessment perceive more support from their entrepreneurial environment (Virginia et al., 2024). This perception can increase their achievement motivation, foster entrepreneurial passion, and avoid the negative impact of individual entrepreneurial failure learning (Zhou and Wang, 2019). When individuals perceive support from family, school, friends, and social environment institutions, their achievement motivation increases, leading to a stronger entrepreneurial intention. Conversely, when individuals perceive hindrances in their environment, their motivation for achievement decreases, resulting in a decline in entrepreneurial intention. Achievement motivation plays a role as a “bridge” between perceived entrepreneurial environment and entrepreneurial intention. The perceived entrepreneurial environment affects the internal factors of the individual, while the entrepreneurial environment (external factors) such as social support, government policies, and infrastructure will play a role in the individual’s achievement motivation (internal factors).

The perceived entrepreneurial environment refers to an individual’s perception of external factors that affect entrepreneurial activities. Entrepreneurial self-efficacy is formed based on the perception of factors within the entrepreneurial environment. When an individual is supported by an objective environment, their sense of security is enhanced, and their sense of fear is eliminated, and this promotes the formation of entrepreneurial self-efficacy. Furthermore, the environment in which the individual is located leads to differences in the growth of entrepreneurial ability. College students, in particular, are in a transitional stage from school to society, and the differences in the entrepreneurial environment may lead to differences in their entrepreneurial knowledge and ability. The diversified driving mechanism of entrepreneurial growth believes that a good entrepreneurial environment can effectively promote the development of entrepreneurial ability, which can in turn enhance the self-efficacy of entrepreneurship and promote the improvement of entrepreneurial intention. Therefore, the subjective environment (perceived entrepreneurial environment) will not only affect the individual entrepreneurial intention through intrinsic psychological motivation (achievement motivation), but also affect the entrepreneurial intention through individual characteristics (entrepreneurial self-efficacy).

In addition, this study found that the mediating effects of achievement motivation and entrepreneurial self-efficacy did not differ significantly. Through the specific path analysis, it was determined that the positive predictive effect of perceived entrepreneurial environment on achievement motivation was greater than that of entrepreneurial self-efficacy. In contrast, the positive predictive effect of entrepreneurial self-efficacy on entrepreneurial intention was greater than that of achievement motivation. The possible explanation for this result is that the perceived entrepreneurial environment directly affects an individual’s achievement motivation, while entrepreneurial self-efficacy is closely related to entrepreneurial intention. An individual’s achievement motivation is formed by perceiving the interaction of the entrepreneurial environment, which promotes the improvement of their entrepreneurial self-efficacy. Therefore, perceived entrepreneurial environment is more closely related to achievement motivation. Moreover, since perceived entrepreneurial environment is an external cause and entrepreneurial self-efficacy is an internal cause, external conditions and factors should function through the individual’s internal psychology. When an individual has stronger confidence in their ability to successfully start a business, their willingness to start a business is stronger. Therefore, entrepreneurial self-efficacy is more closely related to entrepreneurial intention than achievement motivation.

### The chain mediating role of achievement motivation and entrepreneurial self-efficacy

The results of this study show that achievement motivation and entrepreneurial self-efficacy play a mediating role between perceived entrepreneurial environment and entrepreneurial intention, and the total mediation effect is 28.48%. In other words, 28.48% of the effect of perceived entrepreneurial environment on entrepreneurial intention is realized through achievement motivation and entrepreneurial self-efficacy. This highlights that an individual’s entrepreneurial willingness is affected by both external environmental factors and internal factors, and the interaction between the environment and the individual affects the individual’s entrepreneurial intention.

From an individual perspective, the largest mediating effect was found for entrepreneurial self-efficacy with 12.27%, followed by achievement motivation at 10.50%. Achievement motivation can positively predict college students’ entrepreneurial self-efficacy, which is consistent with previous research results (Sun et al., 2013). Entrepreneurial self-efficacy is based on achievement motivation, the higher the achievement motivation, the stronger the independent learning ability, career adaptability, entrepreneurial ability, and career maturity, resulting in a higher sense of entrepreneurial self-efficacy. On the contrary, individuals with low achievement motivation lack internal psychological motivation. They are unwilling to take the initiative to learn entrepreneurship-related knowledge, on the other hand, they also lack career adaptability and entrepreneurial ability, which in turn reduces the sense of entrepreneurial self-efficacy, resulting in a low willingness to start a business.

### Research limitations

Based on the theory of the entrepreneurial intention model, this study aims to examine the relationship between Chinese college students’ perceived entrepreneurial environment and their entrepreneurial intention, along with its underlying mechanisms. The results show that perceived entrepreneurial environment affects Chinese college students’ entrepreneurial intention, which is mediated by achievement motivation and entrepreneurial self-efficacy, and mediated by a chain of achievement motivation → entrepreneurial self-efficacy. These findings not only provide a comprehensive understanding of the psychological mechanism underlying the influence of the perceived entrepreneurial environment on Chinese college students’ entrepreneurial intention but also contribute to the theoretical research on Chinese college students’ entrepreneurial intention. The results of this study include widening employment opportunities for Chinese college students and elevating their employment status, as well as providing theoretical guidance and empirical support to foster, aid, and motivate Chinese college students entrepreneurship.

There are several limitations to this study that should be noted. Firstly, this study only examined the student population of five higher education institutions, with a relatively large proportion of Chinese language students surveyed. Therefore, the standard deviation of each variable in the survey data is relatively small. In future research, the research population will be further expanded to confirm whether this conclusion can be inferred to other groups. Secondly, the perceived entrepreneurial environment is a dynamic and evolving process, and Chinese college students’ entrepreneurial intentions may be influenced by changes in the economic and environmental context. Follow-up studies could explore how changes in the external environment impact entrepreneurial intention over time. Finally, this study controlled for variables such as gender, grade, and family economic level. Future research could investigate the differences between subgroups of Chinese college students based on their family socioeconomic status.

## Conclusion

There were significant correlations between perceived entrepreneurial environment, achievement motivation, entrepreneurial self-efficacy, and Chinese college students’ entrepreneurial intention. Furthermore, the study found that perceived entrepreneurial environment significantly predicted Chinese college students’ entrepreneurial intentions. The mediating effect of achievement motivation and entrepreneurial self-efficacy was also found to be significant in the relationship between Chinese college students’ perceived entrepreneurial environment and entrepreneurial intention. Specifically, there were three intermediary paths identified: the first was the separate mediating role of achievement motivation, the second was the separate mediating role of entrepreneurial self-efficacy, and the third was the chain mediating role of achievement motivation and entrepreneurial self-efficacy. This study has certain theoretical and practical significance, providing specific ideas for universities to carry out innovation and entrepreneurship education. Universities should attach importance to creating a good entrepreneurial atmosphere, and adopt rich club activities and innovation and entrepreneurship competitions to stimulate the achievement motivation of college students, provide opportunities for college students to participate in entrepreneurial activities, enhance their entrepreneurial self-efficacy, and promote their entrepreneurial intentions.

## Data availability statement

The raw data supporting the conclusions of this article will be made available by the authors, without undue reservation.

## Ethics statement

The studies involving humans were approved by the Academic Committee of Quanzhou Normal University. The studies were conducted in accordance with the local legislation and institutional requirements. The participants provided their written informed consent to participate in this study.

## Author contributions

QL: Writing – original draft. YC: Writing – original draft, Writing – review & editing. JL: Writing – original draft. XZ: Writing – original draft.
